# Standardized CT KUB Protocols for Nephrolithiasis: A Retrospective Analysis of Radiation Exposure and Cranial Extent Guidelines

**DOI:** 10.7759/cureus.75743

**Published:** 2024-12-15

**Authors:** Farhan Jarral, Abdelrahman Hamdy, Guleed Mohamed, Rosa Mobayen, Neil Dave, Mohammed Eltawil, Anand Mohan, Osama Abusand, Evripidis Tokidis, Jawaid Akbar

**Affiliations:** 1 Urology, Doncaster Royal Infirmary, Doncaster, GBR; 2 General Surgery, Doncaster Royal Infirmary, Doncaster, GBR; 3 Anesthesiology, Royal Stoke University Hospital, Stoke on Trent, GBR; 4 Otolaryngology, Doncaster Royal Infirmary, Doncaster, GBR; 5 Urology, Chesterfield Royal Hospital, Chesterfield, GBR; 6 Radiology, Doncaster Royal Infirmary, Doncaster, GBR

**Keywords:** clinical audit system, clinical guidelines, computed tomography (ct ), ionizing radiation, ureteric stones

## Abstract

Background and aim

Non-contrast computed tomography of kidneys, ureters, and bladder (CT KUB) is the gold standard radiological imaging for nephrolithiasis. It significantly contributes to the total radiation exposure of a population. This is well known to be linked to increased cancer risk over time and as such should be minimized in line with Ionising Radiation (Medical Exposure) Regulations (IR{ME}R). Previous works have explored a number of avenues to reduce the total radiation exposure such as the cranial extent of the scan; however, at present, there are no formalized guidelines. This study aimed to compare the cranial extent of local CT KUB imaging with previously established thresholds and assess whether total radiation can be reduced through local intervention.

Results

In the first cycle, a total of 102 non-contrast CT KUB scans were included. Of these, 51% (n=52) commenced from the superior border of the T10-T12 vertebral levels, 48% (n=49) commenced above the T10 vertebral level, and only 1% (n=1) started below the T12 vertebral level.

In the second cycle, a total of 105 non-contrast CT KUB scans were assessed. Of these, 21.9% (n=23) commenced above the T10 vertebral level, and 75.2% (n=79) commenced from the superior border of T10-T12 vertebrae. A further 2.9% (n=3) commenced below T12 vertebral level. The findings of this study demonstrate that starting the upper extent of the CT KUB at the T10 vertebral level showed a reduction in radiation dose in millisievert (mSv) delivered to patients while maintaining adequate diagnostic utility.

Conclusion

Limiting the cranial extent of CT KUB imaging to T10 has consistently captured the upper pole of both kidneys across different patient cohorts, including ours, thus making it an effective way of limiting radiation exposure without sacrificing diagnostic accuracy. In order to achieve robust evidence-based guidelines, further studies would be beneficial.

## Introduction

Nephrolithiasis poses a growing challenge for the National Health Service (NHS) in the United Kingdom (UK), with an estimated 10-15% of the population experiencing renal stones during their lifetime and a 50% recurrence rate within five years [1]. Non-contrast computed tomography of the kidneys, ureters, and bladder (CT KUB) is an accessible and highly accurate imaging modality for patients with suspected renal colic with a sensitivity of 95-96% and a specificity of 98% in the diagnosis of nephrolithiasis [2]. There are, however, concerns regarding the use of CT KUB scans as they involve significant exposure to ionizing radiation, with estimates indicating that such scans contribute to over 70% of total radiation doses from medical procedures [3]. In the case of urolithiasis, this poses two problems. First, a significant proportion of patients are young, and second, the risk of recurrent stones is 50% in five to 10 years and 75% in 20 years [4]. One can therefore assume that patients may require multiple scans during their lifetime to assess disease recurrence and/or interval scans prior to intervention thus incurring a greater cumulative radiation exposure. It is therefore important to establish strategies to minimize radiation exposure while not detracting from diagnostic benefits. One strategy to reduce radiation exposure explored in previous works was implementing guidelines stating anatomical landmarks for the cranial extent of CT KUB scans. This method could limit over-scanning and hence reduce unnecessary radiation exposure without any reduction in diagnostic accuracy.

In the initial part of this study, data were gathered with regard to the cranial extent of CT KUB and the actual level at which the kidneys commenced. The second part of the study recommenced a specific level (T10) at which all scans commenced and measured how much radiation was theoretically minimized and whether the diagnostic benefit of the scan was affected due to the change.

## Materials and methods

We undertook a retrospective analysis of non-contrast CT KUB in a District General Hospital in the United Kingdom. The first cohort consisted of 102 patients who underwent non-contrast CT KUB scans for suspected renal calculi during a one-week period in July 2022. Following data collection, the PACS imaging system was utilized to individually review each scan and assess the vertebral level at which the scan commenced and the vertebral level at which the superior pole of the kidneys began. The effective radiation dose per scan in millisievert (mSv) was calculated by multiplying the dose length product (DLP) by a factor of 0.015. Data were extrapolated to Microsoft Excel (Redmond, WA: Microsoft Corp.). Following the initial phase, the findings were presented to the urology and radiology departments, and a new protocol was devised to commence CT KUB scans from the T10-T12 vertebral level. In the second cycle, 105 scans were reviewed in the same manner and the parameters measured were as follows: the number of scans commencing from T10-T12, the level at which kidneys were fully included, and radiation dose per scan.

Inclusion and exclusion criteria

The inclusion criteria for this study were adult patients who underwent non-contrast CT KUB scans for suspected renal colic. The exclusion criteria included any scans that were not non-contrast CT KUB, such as ultrasound (USS), MRI, or contrast CT scans, as well as any CT KUB scans performed for indications other than renal colic.

## Results

All raw data were collated into Microsoft Excel where it was analyzed, as presented in Table 1 for the first cycle (2022). The raw data for the post-intervention cycle (2024) are presented in Table 2.

**Table 1:** Raw data from cycle 1 (2022). CTDI: computed tomography dose index; DLP: dose length product; mGy: milligray; mGycm: milligray centimeter; mSv: millisievert; Y: yes; N: no

1. Vertebral level at which kidneys fully included	2. Vertebral level at which scan commenced	3. Patient dose values as in CTDI (vol) mGy topogram (liters)	4. Patient dose values as in CTDI (vol) mGy (liters)	5. Endpoint pubic symphysis (Y/N)	6. Patient dose values as in DLP mGycm	7. Effective radiation dose (mSv)
Not fully visualized	L1	0.13	4.45	Y	165	2.475
L1	T10	0.03	9.51	Y	464	6.96
L1	T10	0.03	9.09	Y	415	6.225
L1	T10	0.01	2	Y	90.2	1.353
T11	T10	0.13	8.57	Y	428	6.42
T11	T10	0.01	6.35	Y	272.5	4.0875
T11	T10	0.03	9.85	Y	426	6.39
T11	T10	0.01	8.49	Y	369.3	5.5395
T12	T10	0.03	4.69	Y	205	3.075
T12	T10	0.03	11.8	Y	563	8.445
T12	T10	0.01	3.32	Y	132.9	1.9935
T12	T10	0.01	10.47	Y	484.2	7.263
T12	T10	0.03	5.76	Y	137	2.055
T12	T10	0.01	4.67	Y	197.9	2.9685
T12	T10	0.03	5.39	Y	249	3.735
T12	T10	0.03	4.83	Y	244	3.66
T12	T10	0.03	7.87	Y	311.1	4.6665
T12	T10	0.03	8.05	Y	396	5.94
T12	T10	0.01	10.73	Y	429.7	6.4455
T12	T10	0.01	7.37	Y	310.1	4.6515
T12	T10	0.03	7.58	Y	321	4.815
T12	T10	0.03	4.69	Y	205	3.075
L1	T11	0.03	8.05	Y	363	5.445
L1	T11	0.03	3.37	Y	122.2	1.833
L1	T11	0.03	6	Y	235	3.525
L1	T11	0.03	5.58	Y	236	3.54
L2	T11	0.03	5.86	Y	268	4.02
T11	T11	0.13	6.08	Y	231	3.465
T12	T11	0.03	7.46	Y	307	4.605
T12	T11	0.03	3.77	Y	28.8	0.432
T12	T11	0.03	6.84	Y	271	4.065
T12	T11	0.03	6.47	Y	303.9	4.5585
T12	T11	0.03	6.7	Y	293	4.395
T12	T11	0.03	9.51	Y	393	5.895
T12	T11	0.03	4.55	Y	198	2.97
T12	T11	0.01	3.09	Y	122	1.83
T12	T11	0.03	5.85	Y	225.1	3.3765
T12	T11	0.03	35.78	Y	1522.9	22.8435
T12	T11	0.13	16.85	Y	705	10.575
T12	T11	0.13	11.3	Y	460	6.9
T12	T11	0.03	5.58	Y	232	3.48
T12	T11	0.03	7.46	Y	307	4.605
T12	T11	0.01	4.67	Y	197.9	2.9685
L1	T12	0.03	3.75	Y	144	2.16
L1	T12	0.03	4.92	Y	207	3.105
L1	T12	0.03	8.38	Y	318	4.77
L1	T12	0.03	3.46	Y	139	2.085
L1	T12	0.03	6.97	Y	154.1	2.3115
T12	T12	0.03	6.64	Y	277	4.155
T12	T12	0.03	3.89	Y	155	2.325
T12	T12	0.03	5.67	Y	208	3.12
T12	T12	0.13	7.88	Y	295	4.425
T12	T12	0.03	6.84	Y	271	4.065
T11	T6	0.03	17.7	Y	905	13.575
T11	T6	0.03	18.6	Y	994	14.91
L1	T7	0.03	10.1	Y	472	7.08
T11	T7	0.01	5.62	Y	269.3	4.0395
T11	T7	0.03	9.37	Y	483	7.245
T11	T7	0.01	3.26	Y	155.4	2.331
T11	T7	0.03	9.18	Y	434	6.51
T12	T7	0.04	32.18	Y	1702	25.53
T12	T7	0.03	11	Y	618	9.27
L1	T8	0.03	21.9	Y	1199	17.985
L1	T8	0.03	9.3	Y	451	6.765
T10	T8	0.03	6.13	Y	261.3	3.9195
T11	T8	0.03	12	Y	530	7.95
T11	T8	0.03	5.11	Y	260	3.9
T12	T8	0.01	5.9	Y	286	4.29
T12	T8	0.01	6.02	Y	272.6	4.089
T12	T8	0.13	7.17	Y	358	5.37
T12	T8	0.03	13.1	Y	647	9.705
T12	T8	0.03	12.4	Y	628	9.42
T12	T8	0.03	12.9	Y	652	9.78
T12	T8	0.01	8.32	Y	310.1	4.6515
L1	T9	0.03	5.9	Y	289	4.335
L1	T9	0.01	3.12	Y	151.7	2.2755
T11	T9	0.03	15.6	Y	737	11.055
T11	T9	0.01	4.95	Y	213.7	3.2055
T11	T9	0.01	6.63	Y	301.1	4.5165
T11	T9	0.03	6.65	Y	320	4.8
T11	T9	0.03	6.65	Y	320	4.8
T11	T9	0.01	6.58	Y	291.8	4.377
T11	T9	0.03	7.64	Y	350	5.25
T11	T9	0.01	3.94	Y	181.7	2.7255
T12	T9	0.03	6.51	Y	310	4.65
T12	T9	0.01	5.03	Y	236.1	3.5415
T12	T9	0.03	6.58	Y	208	3.12
T12	T9	0.13	6.08	Y	258	3.87
T12	T9	0.01	3.99	Y	166.5	2.4975
T12	T9	0.13	16.4	Y	720	10.8
T12	T9	0.03	7.64	Y	379	5.685
T12	T9	0.01	3.77	Y	190.1	2.8515
T12	T9	0.01	8.6	Y	383.8	5.757
T12	T9	0.01	4.5	Y	215.1	3.2265
T12	T9	0.01	8.6	Y	383.8	5.757
T12	T9	0.01	5.68	Y	377	5.655
T12	T9	0.03	3.75	Y	672	10.08
T12	T9	0.01	5.39	Y	236.8	3.552
T12	T9	0.01	8.21	Y	394.6	5.919
T12	T9	0.03	4.95	Y	238.9	3.5835
T12	T9	0.01	3.99	Y	166.5	2.4975
T12	T9	0.13	6.08	Y	258	3.87

**Table 2:** Raw data from post-intervention cycle (2024). CTDI: computed tomography dose index; DLP: dose length product, mGy: milligray; mGycm: milligray centimeter; mSv: millisievert; Y: yes; N: no The effective dose in mSv was calculated by multiplying DLP by a factor of 0.015.

1. Vertebral level at which kidneys fully included	2. Vertebral level at which scan commenced	3. Patient dose values as in CTDI (vol) mGy topogram (liters)	4. Patient dose values as in CTDI (vol) mGy (liters)	5. Endpoint pubic symphysis (Y/N)	6. Patient dose values as in DLP mGycm	7. Effective radiation dose (mSv)
L1	T12	0.06	3.96	Y	69.39	1.04085
L2	L1	0.01	7.48	Y	294.7	4.4205
Not fully visualized	L1	0.01	3.6	Y	152.7	2.2905
T12	T10	0.03	5.81	Y	251	3.765
T12	T10	0.03	7.4	Y	344	5.16
T12	T10	0.03	7.17	Y	283	4.245
T12	T10	0.03	4.55	Y	191	2.865
T12	T10	0.01	6.24	Y	289.8	4.347
T12	T10	0.03	5.53	Y	235	3.525
T12	T10	0.03	3.2	Y	146.5	2.1975
T11	T10	0.03	4.22	Y	166.6	2.499
T12	T10	0.03	4.72	Y	210.7	3.1605
L1	T10	0.03	6.75	Y	305	4.575
T12	T10	0.03	8.38	Y	363	5.445
T11	T10	0.01	5.45	Y	235.7	3.5355
T12	T10	0.03	11.1	Y	470	7.05
T12	T10	0.01	6.19	Y	263	3.945
L1	T10	0.03	6.93	Y	319	4.785
T11	T10	0.03	9.77	Y	443	6.645
T12	T10	0.03	6.09	Y	270	4.05
L1	T10	0.03	5.47	Y	243	3.645
T12	T10	0.03	6.7	Y	277	4.155
T11	T10	0.03	17.5	Y	729	10.935
T12	T10	0.03	4.87	Y	214	3.21
T11	T10	0.01	7.76	Y	384.1	5.7615
T12	T10	0.03	4.97	Y	211	3.165
T11	T10	0.03	6.46	Y	269	4.035
T11	T10	0.01	6.13	Y	284.4	4.266
T12	T10	0.01	4.84	Y	211.1	3.1665
T11	T10	0.03	5.12	Y	219.3	3.2895
T11	T10	0.03	8.45	Y	370	5.55
T12	T10	0.01	5.73	Y	261.5	3.9225
T12	T10	0.01	4.95	Y	215.5	3.2325
T12	T10	0.03	4.59	Y	208	3.12
T12	T10	0.03	6.98	Y	315	4.725
T12	T10	0.03	6	Y	273	4.095
T11	T10	0.03	10.9	Y	460	6.9
T12	T11	0.03	12	Y	526	7.89
L1	T11	0.03	3.06	Y	114.9	1.7235
T12	T11	0.06	2.83	Y	66.26	0.9939
L1	T11	0.03	4.53	Y	177.4	2.661
T11	T11	0.03	9.34	Y	376	5.64
T12	T11	0.03	5.57	Y	211	3.165
T12	T11	0.03	7.46	Y	320	4.8
L1	T11	0.03	8.66	Y	376	5.64
T12	T11	0.03	8.87	Y	377	5.655
L1	T11	0.01	4.05	Y	261.8	3.927
T12	T11	0.06	3.37	Y	81.2	1.218
T12	T11	0.03	7.52	Y	341	5.115
T12	T11	0.01	8.15	Y	313.8	4.707
L1	T11	0.03	8.67	Y	364	5.46
T12	T11	0.03	5.29	Y	228	3.42
T12	T11	0.03	3.29	Y	133.9	2.0085
T12	T11	0.01	3.77	Y	155.4	2.331
T12	T11	0.01	6.52	Y	272.5	4.0875
T12	T11	0.01	4.89	Y	196.3	2.9445
T11	T11	0.03	8.8	Y	362	5.43
L1	T11	0.03	22.8	Y	934	14.01
T12	T11	0.03	9.93	Y	401	6.015
T12	T11	0.01	4.22	Y	169.1	2.5365
T12	T11	0.01	4.4	Y	189	2.835
T12	T11	0.03	13	Y	511	7.665
L1	T11	0.01	5.23	Y	215.2	3.228
T12	T11	0.03	6	Y	238	3.57
T12	T11	0.03	3.43	Y	129.6	1.944
L1	T11	0.03	15.6	Y	588	8.82
L1	T12	0.01	3.65	Y	151.9	2.2785
L1	T12	0.03	6.93	Y	273	4.095
L1	T12	0.03	6.81	Y	299	4.485
L1	T12	0.03	4.12	Y	164	2.46
T12	T12	0.01	11.24	Y	454.3	6.8145
L1	T12	0.01	8.15	Y	338.8	5.082
L2	T12	0.06	3.3	Y	179.94	2.6991
L1	T12	0.01	3.22	Y	111.9	1.6785
L1	T12	0.03	7.22	Y	303	4.545
L1	T12	0.06	3.61	Y	83.61	1.25415
L1	T12	0.01	7.82	Y	299	4.485
L1	T12	0.01	4.1	Y	165.9	2.4885
L1	T12	0.03	6.23	Y	254	3.81
t12	T12	0.01	2.4	Y	95.2	1.428
Not fully visualized	t12	0.06	3.37	Y	77.1	1.1565
L2	t12	0.01	3.15	Y	119	1.785
T10	T6	0.01	6.24	Y	319.5	4.7925
T12	T6	0.01	5.29	Y	275.9	4.1385
T12	T6	0.01	8.66	Y	470.3	7.0545
T11	T7	0.03	12.76	Y	643.3	9.6495
L1	T7	0.03	20.7	Y	1165	17.475
T11	T7	0.03	7.52	Y	383	5.745
T12	T7	0.03	19	Y	943	14.145
T11	T7	0.03	14.8	Y	838	12.57
T12	T7	0.03	6.84	Y	361	5.415
L1	T7	0.03	6.47	Y	302.6	4.539
L1	T8	0.01	5.06	Y	277.3	4.1595
T12	T8	0.03	10.8	Y	524	7.86
L1	T8	0.06	3.93	Y	525.7	7.8855
L1	T9	0.03	5.99	Y	273	4.095
T12	T9	0.03	8.73	Y	434	6.51
L1	T9	0.01	5.75	Y	268.2	4.023
T12	T9	0.01	6.3	Y	283.4	4.251
T11	T9	0.03	5.29	Y	245	3.675
L1	T9	0.03	6.23	Y	275	4.125
T11	T9	0.03	6.56	Y	302	4.53
L1	T9	0.03	12	Y	540	8.1
L1	T9	0.03	9.37	Y	467	7.005
T12	T9	0.03	5.96	Y	250	3.75

First cycle (pre-intervention)

**Figure 1:**
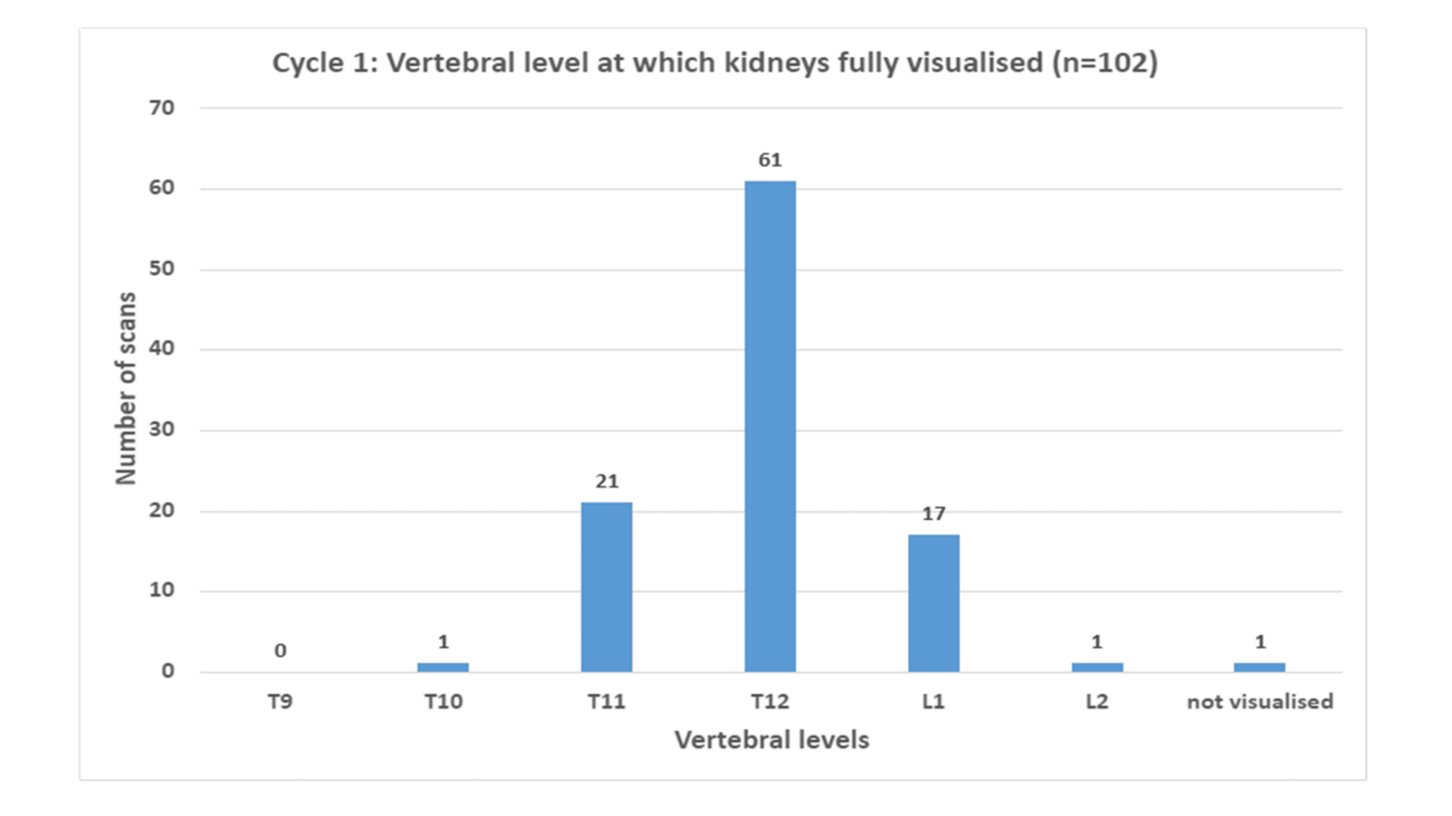
Chart demonstrating the level at which kidneys are fully visualized in cycle 1 (pre-intervention).

In the initial cycle in 2022, a total of 102 non-contrast CT KUB scans were included. We examined the vertebral level at which the upper pole of the kidney was visualized. For all of the studies, 99% (n=101) of the kidneys were fully visualized from the vertebral level of T10 or below. A total of 98% (n=100) of kidneys were fully visualized from T11, whereas at the vertebral level of T12, this number was 77.5% (n=79). One scan commenced from the L1 vertebral level and did not fully visualize the kidneys (Figure 1).

**Figure 2:**
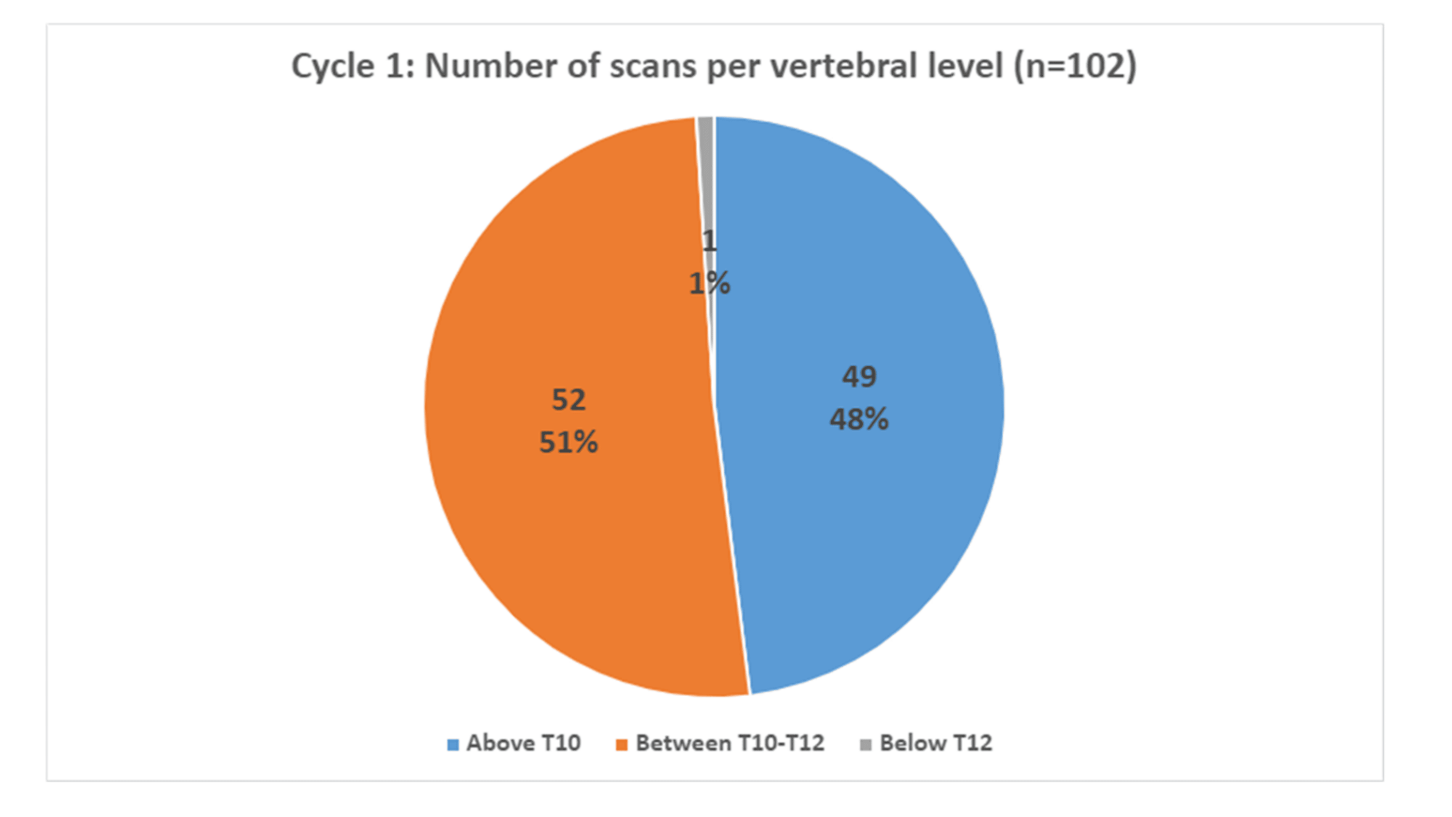
Chart to demonstrate vertebral level at which scans start in cycle 1 (pre-intervention).

With regards to the cranial level at which the scans commenced, 48% (n=49) of scans commenced above the T10 vertebral level. The highest vertebral level of scan commencement was the superior border of the T6. Furthermore, 51% (n=52) of scans commenced between the superior border of T10 and the inferior border of T12. A further 1% (n=1) of scans started below T12 vertebral level (Figure 2).

**Figure 3:**
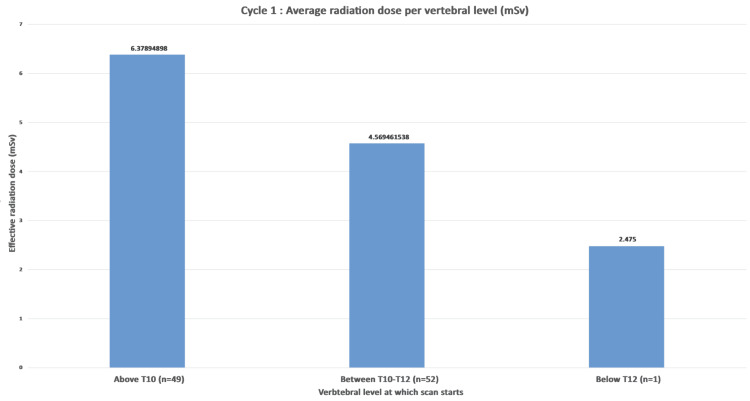
Cycle 1: average exposure to radiation per vertebral level groups (n=102). The effective dose in mSv was calculated by multiplying DLP by a conversion factor of 0.015. mSV: millisievert; DLP: dose length product

On examination of the radiation exposure, the average effective radiation dose for scans commencing above T10 was 6.37 mSv. For scans starting from the superior border of T10 to the inferior border of T12, the average dose was 4.56 mSv. Scans performed below the vertebral level of T12 had an average radiation dose of 2.47 mSv (Figure 3).

Second cycle (post-intervention)

**Figure 4:**
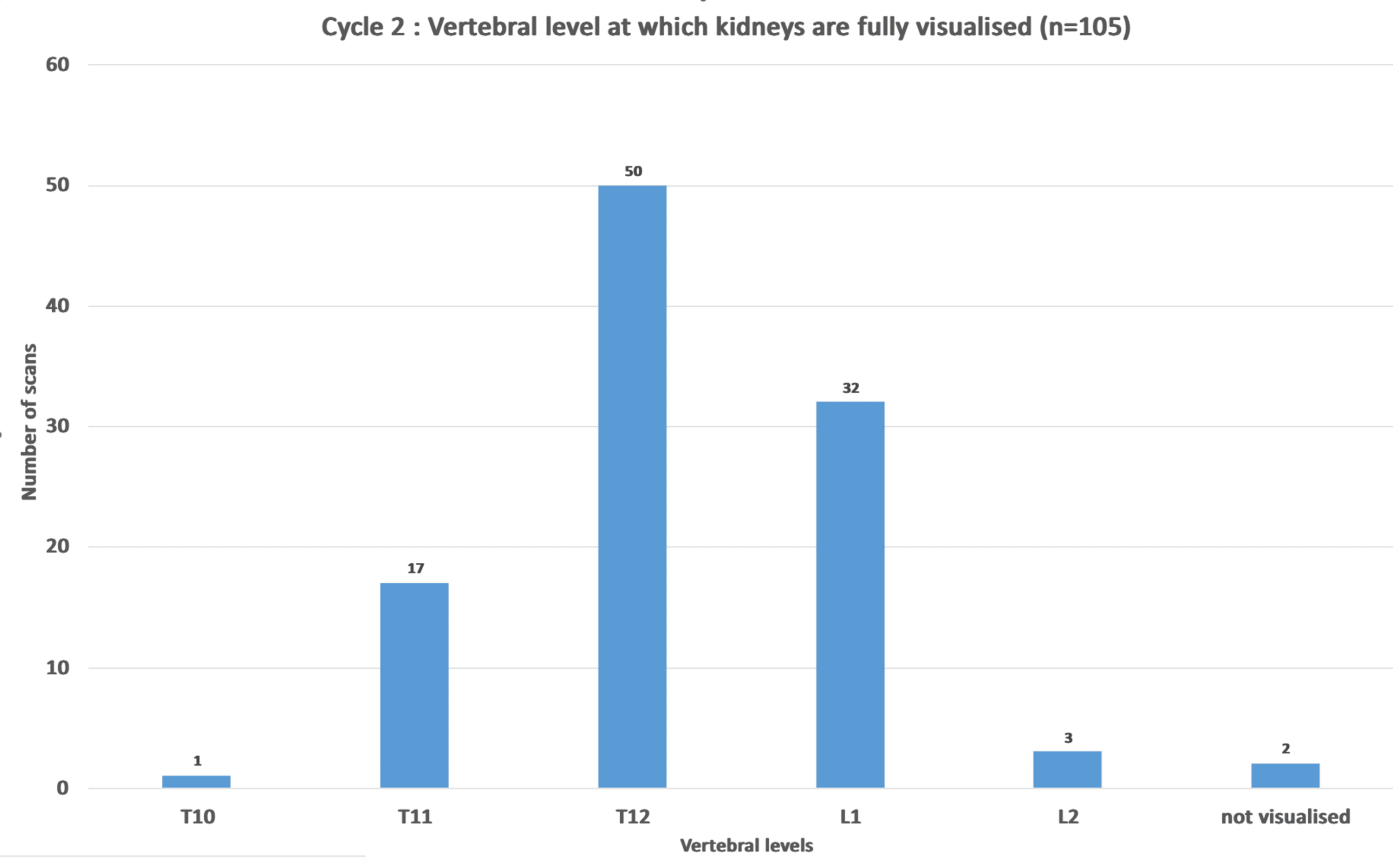
Chart demonstrating vertebral level at which kidneys are visualized in cycle 2 (post-intervention).

In the post-intervention (second cycle), there were a total of 105 non-contrast CT KUB scans. A total of 98.1% (n=103) studies showed kidneys fully visualized from vertebral level of T10 or below. In 97.1% (n=102) of CT studies, it was shown that the kidneys were fully visualized at or below T11 vertebrae. In 80% (n=85), the kidneys were visualized at T12 vertebrae or below. For 1.9% (n=2) of studies, the kidneys were not fully visualized but this was due to the scan commencing too low (Figure 4).

**Figure 5:**
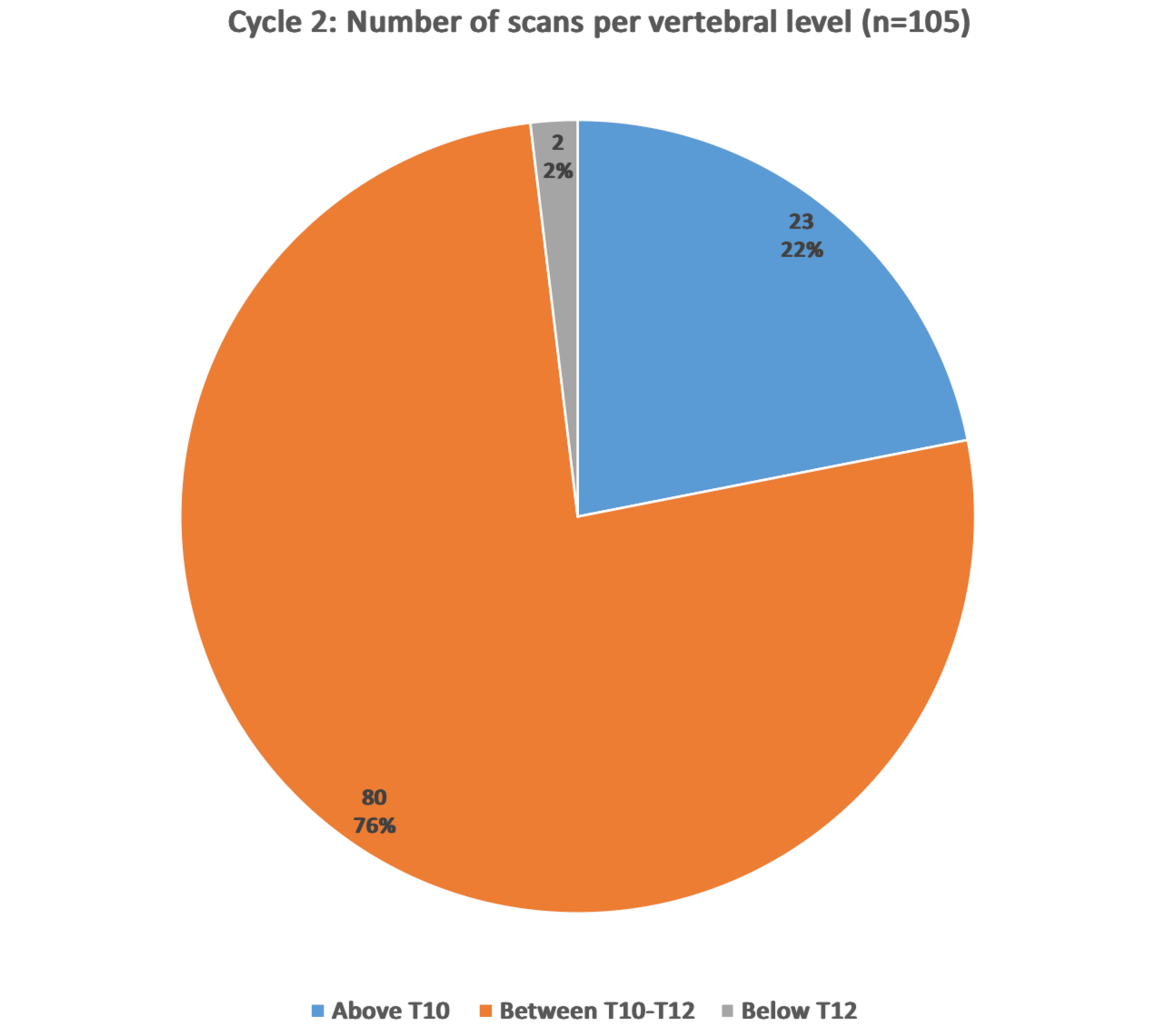
Chart to demonstrate vertebral level at which scans start in cycle 2 (post-intervention).

The post-intervention data collection demonstrates that 21.9% (n=23) of scans commenced above the vertebral level of T10, highest at the vertebral level of T6. The majority of scans, 76.19% (n=80), started from the superior border of T10 to the inferior border of T12 level. A further 1.9% (n=2) of scans began from vertebral level below T12 (Figure 5).

**Figure 6:**
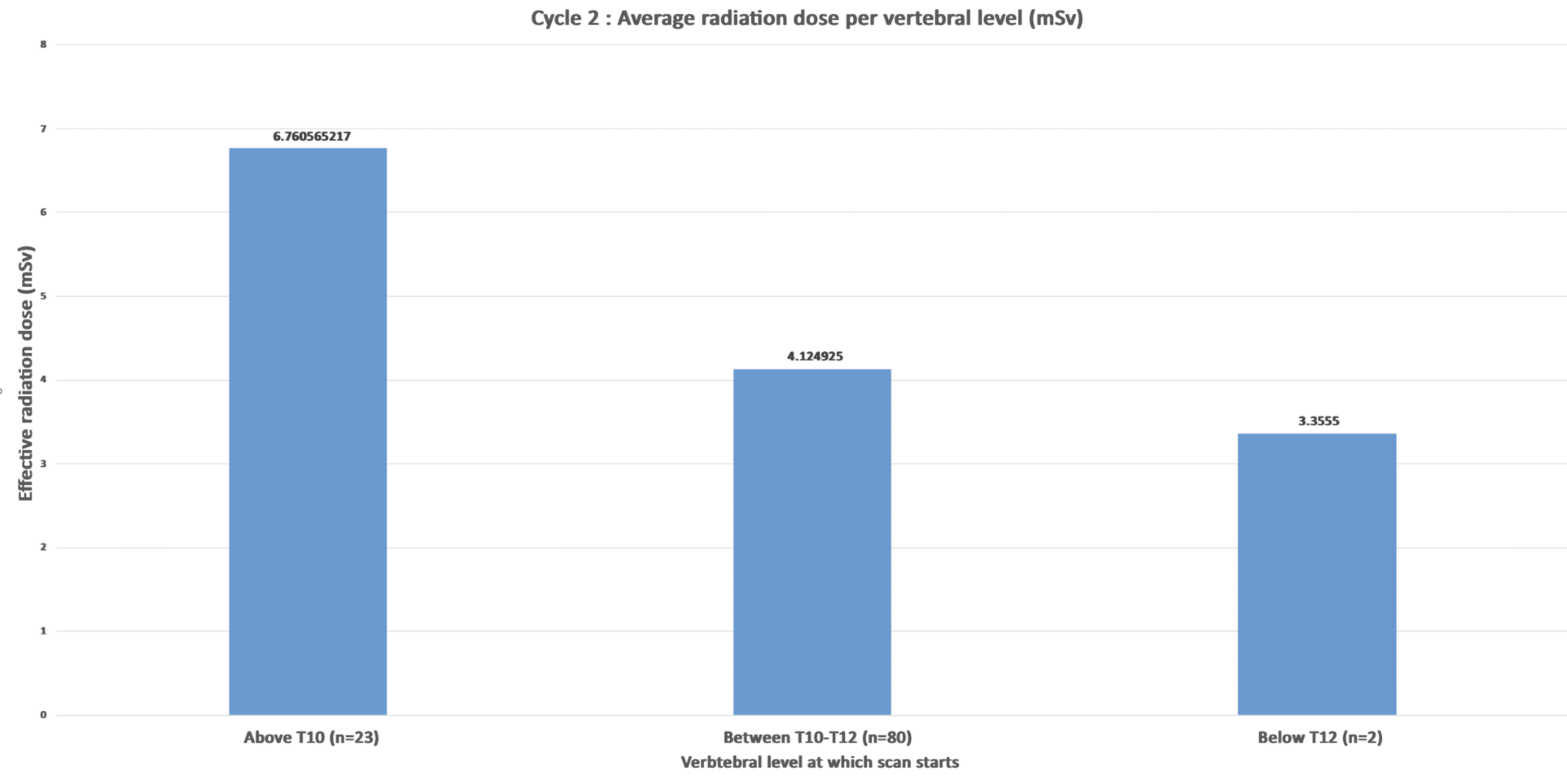
Cycle 2: average exposure to radiation (mSv) per vertebral level groups (n=105). mSv: millisievert; DLP: dose length product The effective dose in mSv was calculated by multiplying DLP by a conversion factor of 0.015.

The average effective radiation dose for scans commencing above T10 was 6.76 mSv. Radiation for scans starting from the superior border of T10 to the inferior border of T12 was 4.12 mSv, and for scans commencing from below T12 was 3.35 mSv (Figure 6).

## Discussion

The standard radiation dose from a CT KUB in literature is stated to be 4.3-14 mSv [5]. As medical professionals and in line with the Ionising Radiation (Medical Exposure) Regulations (IR{ME}R), one has a responsibility to minimize unintended, excessive, or incorrect medical exposures [6]. It is therefore incumbent to continuously evaluate practice to enable the development of standardized, safer, and more efficient strategies to minimize radiation exposure while maintaining diagnostic benefits. The effective radiation dose was calculated for this study by multiplying the DLP by a factor of 0.015 based on online radiology tools [7].

Anatomically, the kidneys are retroperitoneal structures that are typically found between the T12 and L3 vertebral levels [8]. It would be reasonable to utilize these vertebral levels as a basis to delineate the landmark of the cranial extent of CT KUB scans. The results of this study, however, demonstrated that starting the scan at the T12 vertebral level may risk under-scanning and not visualizing parts of the kidneys, which, in turn, causes further exposure to radiation by requiring repeat scans. This study showed that both kidneys were fully visualized from the T10 vertebral level in 98.6% (n=204) of cases. The remaining 1.4% (n=3) of scans did not fully visualize the kidneys due to commencing the scan too low (one at T12 vertebral and two at L1 vertebral level), therefore the upper extent of the kidney could not be established. From the T11 vertebral level, 97.6% (n=202) of scans fully visualized the kidneys, while from the T12 vertebral level, only 79.2% (n=164) of scans fully visualized the kidneys. The first cycle results also demonstrate a discrepancy between the cranial extents of the scans, which is evident as some scans commenced between one to six vertebral levels above the radiological superior aspect of the kidneys, the highest of which was recorded at the T6 vertebra. Of the 102 patients scanned in the first cycle, 48% (n=49) were scanned above T10. This may be an indication of a lack of standardized scanning protocol which has also been demonstrated by Ahmad et al. in their audit of 100 CT KUB scans, of which 76% commenced above T10, adding no diagnostic benefit [9]. In the post-intervention cycle of this study (cycle 2), 21.9% (n=23) were scanned above the T10 vertebral level compared to 49.5% (n=50) in the pre-intervention cycle. This demonstrates a reduction in the proportion of scans that were commenced higher than necessary compared to the initial cycle.

One can question whether the T10 or T11 vertebral levels would be more suited to form the cranial start point. Though the T10 vertebral level may expose patients to potentially increased radiation, starting at the T11 vertebral level has an increased risk of under-scanning.

With regards to the effective radiation dose at the various levels, combined data from both cycles demonstrate individuals with CT scans commencing above the level of T10 vertebrae receive an effective dose of radiation equalling 6.5 mSv on average. However, individuals with scans commencing between T10 and T12 vertebrae receive a dose equalling 4.3 mSv on average. These findings suggest that scanning above the level of the T10 vertebra adds no diagnostic value while exposing individuals to an added dose of radiation. This is important as patients with nephrolithiasis, as aforementioned, often have recurrent stones and thus will require repeat scans. Limiting the exposure to radiation by only scanning areas of diagnostic benefits can reduce their cumulative radiation exposure. Ferrandino et al. investigated the cumulative radiation exposure from CT KUB scans among patients with kidney stones, particularly focusing on those who undergo frequent imaging. Their results revealed that the median dose of radiation individuals were subjected to was 29.7 mSv with nearly one-fifth of patients reaching cumulative doses above 50 mSv, a level linked with increased cancer risk over time. This heightened exposure raises specific concerns for younger patients who may need repeated scans throughout their lives. To address these risks, the study advocates the use of lower-dose imaging protocols or, wherever possible, alternative methods like ultrasound to balance diagnostic needs with patient safety over time [10].

Though there is sparse literature studying this topic, some previous works have been conducted. Maguire and Gray assessed the optimal cranial extent for CT KUB scans, focusing on reducing unnecessary radiation. Their study analyzed the coverage of the kidneys starting at various vertebral levels, emphasizing that initiating scans at T10 adequately captured the kidneys' upper poles in all patients. By limiting scans to T10 instead of extending higher, they achieved an approximately 16% reduction in radiation dose. Additionally, they reported that starting at T10 reduced exposure to unnecessary thoracic anatomy [11].

Uldin et al. explored an even more conservative approach by suggesting T11 as a starting point for CT KUB scans. Their findings revealed that using T11 resulted in a substantial 62.4% reduction in mean scanning length between the two phases of the study, effectively decreasing radiation exposure without compromising diagnostic integrity. This research indicates that T11 may serve as a safer alternative to T10 [12]. Similarly, Cavenagh et al. indicated that T10 serves as the upper boundary for CT KUB imaging to prevent underscanning. However, they also noted that utilizing the vertebral level of T11 would also ensure that both kidneys are completely captured [13].

In line with Uldin et al., Ghoshal and Gaikstas assessed 88 patients and found that 89.8% of the scans exceeded the target of less than 10% over scanning above the highest kidney, resulting in an average over-scan of 16.4%. Consistent with the aforementioned study, their findings suggest that beginning scans at the upper border of the T11 vertebra could effectively minimize unnecessary radiation while ensuring comprehensive imaging of the kidneys [12,14].

Marrying the findings of this study with the findings of previous studies, it is evident that a robust scanning protocol is required. There is some debate as to which vertebral level should form the cranial limit of CT KUB. Given that the kidneys are always fully visualized at T10, it is certain that scans should not extend above this point, and, in fact, it may be argued that T10 should form the cranial extent.

Limitations

There are several potential limitations of this study. Firstly, the scans were reviewed by multiple individuals thus there is a risk of inter-observer variability as to the exact cranial extent measured. Secondly, factors such as patient body mass index (BMI), height, etc., have not been taken into account with regard to measuring radiation. Thirdly, as the CT scan procedures were not under direct supervision, adherence to the protocol could not be ensured. Lastly, the patients in both cycles were not case-matched for age, height, and BMI.

## Conclusions

Given the high prevalence of patients with nephrolithiasis, CT KUB scans are increasingly common. Additionally, a large cohort of patients are recurrent stone formers and, thus, require repeated scans and subsequent exposure to ionizing radiation. This exposure as discussed is potentially harmful over their lifetime. A standardized approach is needed with regard to performing a CT KUB in order to limit excess ionizing radiation while maintaining its diagnostic value. From the few studies relating to this topic, there is some debate as to whether the cranial extent of the scan should be at the T10 or T11 vertebral level. Though the T11 vertebral level certainly may expose patients to less radiation, this study found that there are rare instances of the upper level of kidneys commencing at T10 radiologically. For this reason, the T10 vertebral level may result in an acceptable balance between radiation dose and maintaining scan adequacy.

Though a number of studies and audits have demonstrated local protocol implementation with a successful reduction in radiation doses, a standardized guideline is not available to allow national implementation. In order to achieve evidence-based guidelines with regard to the cranial extent of CT KUB scans, larger multi-centered studies would be beneficial.
